# 23 Wound Characteristics and Other Predictive Factors of Hypertrophic Scarring in Pediatric Burns

**DOI:** 10.1093/jbcr/iraf019.023

**Published:** 2025-04-01

**Authors:** Jessica Willoughby, Kathy Prelack, Delaney Moslander

**Affiliations:** Shriners Children’s Boston; Shriners Children’s Boston; Shriners Children’s Boston

## Abstract

**Introduction:**

Though generally accepted, it is not a definitive rule that burn injuries under 10% TBSA that heal in less than 21 days will have no scarring. The aim of this study was to assess what factors impact the likelihood of a child developing hypertrophic scarring secondary to a burn injury healed under 21 days compared to those who healed over 21 days.

**Methods:**

A retrospective study of 2,016 pediatric patients with burn injuries under 10% TBSA from a five-year period at a single burn center’s outpatient clinic was conducted. Descriptive statistics, Chi-square analysis, and logistic regression were used to describe the patients, compare those who developed scars to those who did not, and identify which factors increased a patient’s odds of developing hypertrophic scarring. A scar was considered present if the patient’s chart included a Modified Vancouver Scar Scale score or if medical notes or photography indicated a raised scar.

**Results:**

Of the patients reviewed, 1,667 patients healed under 21 days, 349 healed over 21 days, and 13.5% of all patients developed hypertrophic scarring. There were three factors that significantly predicted higher odds of developing hypertrophic scarring in both populations: deeper wounds (OR = 13.324, p < 0.001; OR = 0.344, p = 0.018), increased time to healing (OR = 11.819, p < 0.001; OR = 0.254, p < 0.001), and higher %TBSA (OR = 1.497, p < 0.001; OR = 0.860, p = 0.039).

This research indicated that children with Fitzpatrick skin type 6 whose wounds healed in over 21 days were less likely to develop hypertrophic scars than patients with lighter skin tones (OR = 5.426; p = 0.010).

In the under 21 days group, being born female significantly increased the odds of hypertrophic scarring (OR = 1.934; p = 0.014). Additionally in this group, injuries on any part of the upper extremity, anterior trunk, thighs, head or face were significantly less likely to develop hypertrophic scarring than injuries on the palmar hand.

**Conclusions:**

The results of this study are consistent with previous findings that larger and deeper wounds taking longer to heal are more likely to develop hypertrophic scarring. Contrary to previous research, patients with Fitzpatrick skin type 6 had lower odds of developing hypertrophic scarring. This result should be interpreted with caution as only 4.86% of all patients were Fitzpatrick skin type 6. The model accurately predicted scarring in the over 21-days group 64.8% of the time. However, within the under 21-days group, the model was only able to accurately predict development of hypertrophic scarring 5.5% of the time.

**Applicability of Research to Practice:**

The ability to identify children who are at risk for development of hypertrophic scarring allows practitioners to provide interventions sooner to help decrease the sequelae from troublesome scars.

**Funding for the Study:**

N/A

